# United States cattle market location and annual market sales estimate data

**DOI:** 10.1016/j.dib.2025.111877

**Published:** 2025-07-11

**Authors:** Samuel M. Smith, Clayton Hallman, Tom Lindström, Stefan Sellman, Ryan S. Miller, Katie Portacci, Colleen T. Webb, Lindsay M. Beck-Johnson

**Affiliations:** a Department of Biology, Colorado State University, Fort Collins, CO, USA; b Graduate Degree Program in Ecology, Colorado State University, Fort Collins, CO, USA; c USDA APHIS Veterinary Services, Center for Epidemiology and Animal Health, Fort Collins, CO, USA; d Department of Physics, Chemistry and Biology, Division of Theoretical Biology, Linköping University, Linköping, Sweden

**Keywords:** Animal science, Computational biology, Livestock demography, Cattle shipment networks, Livestock disease modeling, Movement, Market sales

## Abstract

Cattle markets, where livestock producers may buy and sell cattle and calves, act as major hubs in the shipment network that connect cattle populations across the United States (U.S.). Cattle markets can then provide insight into the integration of the U.S. cattle industry, thus informing how regional price fluctuations may influence cattle prices nationally. Despite biosecurity measures and regulatory compliance from livestock markets, commingling and re-distribution of animals from multiple sources may elevate the risk of disease spread and make tracing animal movements more complex, which could pose significant challenges if a transboundary animal disease (TAD) were introduced into the U.S. Therefore, knowing the size and location of cattle markets in the U.S. is critical to understanding cattle industry market dynamics and enhancing pandemic scenario modeling efforts. In this article, we present a list of cattle markets, their locations, and estimated quarterly cattle sales. We compiled a list of 1619 known cattle markets with and without market sales data from 1131 counties across the U.S. from 2012–2016. To estimate unknown market sales data, we fit a spatial autoregressive lag model to annual county-level market sales data and used the fit to predict annual sales in counties that lacked sales information. County-level sales data provide important insight into the structure of the U.S. cattle industry. The dataset can be used to improve national-scale cattle movement models, livestock disease models, and inform TAD surveillance efforts.

Specifications Table

**Table:** 

Subject	Mathematical modeling (Data Science)
Specific subject area	Livestock movements and trade
Type of data	Comma-separated values file
How the data were acquired	Cattle market locations were collected from Carroll and Bansal [1], United States Department of Agriculture’s (USDA) Federally Approved Market List, Grain Inspection Service (GIPSA), and Livestock Market Association (LMA) and market sales data were added using the USDA’s Agricultural Marketing Service (AMS) where available [2]. We calculated average yearly cattle market sales for 266 counties for which market sales data from 2012–2016 were available. A spatial model was fit to known annual county market sales data using the *lagsarlm()* function from R package *spdep* [3], which was then used to estimate annual county-level market sales for the remaining counties without pre-existing market data*.*
Data format	Raw and analyzed
Description of data collection	We merged Carroll and Bansal [1]’s cattle market location list with market lists from GIPSA, LMA, and USDA’s Federally Approved Market Association [4,5], removed duplicated entries, and verified markets with Google Street View. After removing duplicates and verifying market locations, we geocoding market locations with ArcGIS. We also added 2012–2016 sales information from the USDA’s AMS [2].
Data source location	• Country: United States of America • USDA Animal and Plant Health Inspection Service (APHIS) Federally Approved Market List • USDA Grain Inspection Service (GIPSA) • USDA Agricultural Marketing Service (AMS) • Livestock Market Association (LMA)
Data accessibility	Repository name: Dryad Digital Repository Data identification number: doi:10.5061/dryad.cfxpnvxg7 Direct URL to data: https://datadryad.org/stash/share/RxD26wfIKo8VqcZXNO8bE37ELPOuq-T66YJE-XTOECc
Related research article	S. Sellman, L.M. Beck-Johnson, C. Hallman, R.S. Miller, K.A.O. Bonner, K. Portacci, C.T. Webb, T. Lindström, Modeling U.S. cattle movements until the cows come home: Who ships to whom and how many?, Comput Electron Agric. 203 (2022).

## Value of the Data

• The National Agricultural Statistical Survey (NASS) Census of Agriculture does not provide cattle market demography data [6], so we provide the first comprehensive and validated list of cattle market locations and county-level sales data, which is necessary to accurately model cattle movements across the United States.
• These data are useful to researchers interested in cattle market demography and movements across the United States, whether their research is related to TADs or not.
• These data provide insight into how the United States livestock industry is geographically structured. A list of cattle market locations and county-level sales data can inform economic models of the cattle industry, disease surveillance efforts, and enhance emergency disease preparedness by facilitating tracing efforts if a TAD were detected. Yearly volume estimates for cattle markets provides insight into the potential relative importance of any given market to controlling price fluctuations or disease spread. Both cattle market location and volume estimates are critical to modeling cattle movements across the United States.
• These data are from 2012–2016 and were intended to help provide county-level predictions of cattle movements and disease spread by identifying counties that send and receive high and low volumes of cattle. Our experience suggests that the county-level structure of cattle markets fluctuates less year over year than specific cattle markets, which may have gone in and out of business since these data were collected.

## 1 Objective

We generated these data to improve the cattle shipment network generated by the United States Animal Movement Model (USAMM) [[7], [8], [9]] and predictions of disease spread in the United States Disease Outbreak Simulation (USDOS) [10,11]. This dataset resulted in more accurate USAMM predictions by ensuring that movements through high contact market premises are captured accurately in the shipment networks. USDOS predictions of disease spread also improved due to these more accurate networks because they provided the opportunity for disease spread to occur during the mixing of cattle at markets. Additionally, a comprehensive and validated list of cattle market locations and county-level sales data is more generally valuable as it helps provide a more accurate understanding of how the United States’ cattle industry is geographically structured.

## 2 Data Description

This dataset provides a list of all known markets that sell cattle in the United States in addition to county-level annual cattle sales estimates for 1619 markets from 1131 counties in the United States between 2012 and 2016 [1]. Some markets may sell other species in addition to cattle, but these details are not available in the data sources. Therefore, this dataset does not distinguish between cattle-only markets and those that may sell multiple animal species. More than one market is present in 361 of these counties. Every market can be identified in the dataset with an identification number listed in the premises.Beck-Johnson column.

Values in MarketList_final.csv are comma-separated with 14 columns:

1. RecordID: Unique identifier for each record (matches MarketID column in the Carroll and Bansal de-duplicated market list).
2. premises.CB: Premises designations from Carroll and Bansal [1].
3. premises.Beck-Johnson: Corrected premises designations.
4. FIPS: A five-digit Federal Information Processing Standard (FIPS) code that uniquely identifies county centroids.
5. Notes: Canada (Canadian markets), Horse (Google searches show auction is associated with horses, with no indication of other livestock), No Livestock (where Google searches show auction is associated with goods other than livestock), Insufficient Location Information (Cannot determine county based on available information).
6. name: Market name from source. Different names are present for the same market if sources listed different names.
7. address: The physical location or business address of a market.
8. po: Post Office box.
9. city: City that the market is located in.
10. state: State the market is located in.
11. zip: United States Postal Service Zone Improvement Plan (ZIP) code.
12. zip_ext: ZIP code extension number.
13. source: Market information source.
14. id: Market identification number from source.

Values in Market_Volume_Estimates.csv are comma-separated with four columns:

1. FIPS: A five-digit Federal Information Processing Standard (FIPS) code that uniquely identifies county centroids.
2. number_markets: The number of markets in that county.
3. volume_data: average number of cattle sold per year in that county, where known.
4. estimated_volume: estimated annual cattle sales where data were not available. Known sales data (e.g. from volume_data column) are in this column where available.

A list of all cattle markets in the US with annual volume estimates provides insight into how the cattle industry is structured, how cattle markets could contribute to TAD dynamics, and promote the traceability of cattle in the US.

## 3 Experimental Design, Materials and Methods

**Fig. 1:**
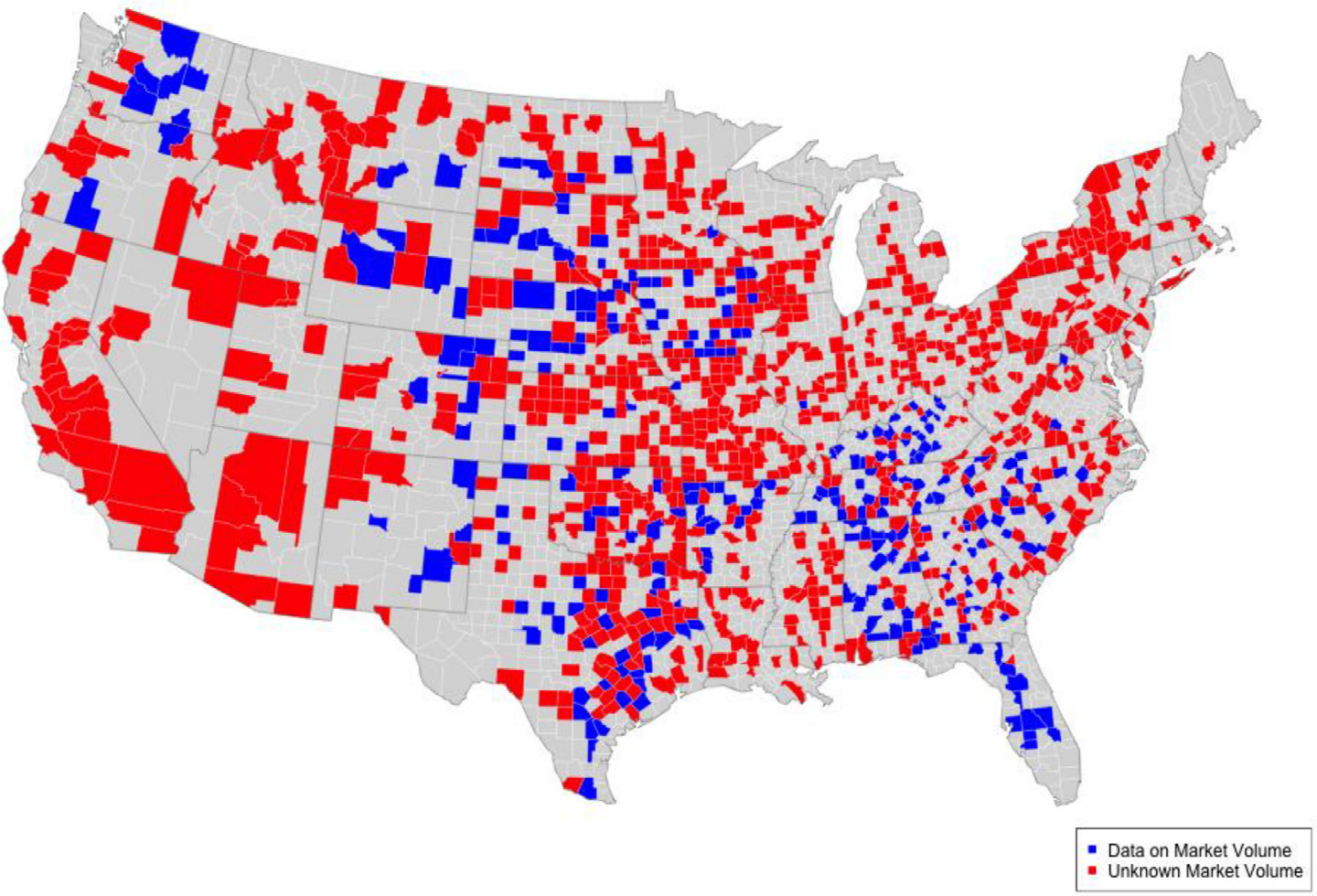
Distribution of counties with market volume data. Blue counties have market volume data. Red counties have known markets, but no data available on volumes. Grey counties do not have any known markets.

We generated a comprehensive list of cattle market locations by merging lists of cattle markets from Carroll and Bansal [1], USDA APHIS’s Federally Approved Market List, USDA Grain Inspection Service, USDA AMS, and LMA websites. After merging these lists we verified individual market locations, removed duplicated markets, and added market sales data of cattle from USDA’s Agriculture Marketing Service (AMS) [2,4,5]. We removed duplicates and verified market locations using Google Street View, which allowed us to ensure that the business address was where the market was physically located. If a market could not be verified using Google Street View, but was on the original list and clearly not a duplicated entry (e.g. shared a business address), we opted to leave the market on the list. We made this choice to ensure that we captured all possible locations where cattle are bought and sold in the United States. Markets were then geocoded in ArcGIS after their locations were verified. By merging cattle market lists from all of the available sources, our protocol was designed to identify every market where cattle are bought and sold in the United States during this time period. Our final market list included 1639 unique market locations. Markets from 266 counties had sales data, but markets in 865 counties lacked this information (Fig. 1). We found no known markets in 1915 counties in the United States. The known cattle market sales data for the 266 counties was recorded daily. AMS provides sales data for cattle markets but does not distinguish between virtual and in person sales. To estimate volume in the 865 counties without sales data, we averaged the total number of animals sold at a market every year and then aggregated these sales data to the county-level. Strong spatial structure was present in county-level sales estimates after aggregation (Moran’s *I* = 0.33, *p* < 0.0001, Table 1). We then used the total number of cattle sold in a county from the 2012 NASS survey and the number of markets present in a given county as covariates to fit a spatial simultaneous autoregressive lag model to annual market volumes from the 266 counties with known markets [[12], [13], [14]]:

(1) y=ρWy+Xβ+γWy+∈

$$\in ∼N\left(0,\;\sigma^{2}I_{n}\right)$$

where y is a *n* x 1 vector of annual average market sales for *n* number of counties with markets, ρand γ are scalar autoregressive parameters, and β is a *k* x 1 vector of parameters. ∈ is a *n* x 1 vector of independently and identically distributed residuals. **X** is a *n* x *k* matrix of *k* spatially lagged covariates. W is a *n* x *n* spatial weight matrix where

(2) $$W_{n_{ij}}=\exp\left(\frac{-d_{ij}}{250\;km}\right)$$

and $d_{ij}$ is the distance between centroids of county i and county j. 250 km is the average distance animals are shipped to markets [15]. The number of markets and annual cattle sales (in head) in each county were the only covariates included in the model and were left unstandardized (Table 2). This model structure allowed for market sales estimates in each county to be strongly influenced by market sales in surrounding counties by weighting each county’s total market volume estimate with the spatial weight matrix, W [[12], [13], [14]].

**Table 1:** Estimated spatial autocorrelation using Moran’s I for both the model’s predicted values (fitted) and the difference between model predictions and observed values (residual). Moran’s I ranges between one and negative one, where one represents perfect spatial autocorrelation, negative one would be perfect negative spatial correlation, and zero is no spatial autocorrelation. Moran’s I standard deviate is a standardized test statistic for Moran’s I.

Type	Moran's I statistic	Expectation	Variance	Moran's I standard deviate	P-value
Data	0.33	−0.0038	0.0012	9.9	2.6e-23
Fitted	0.76	−0.0038	0.0013	21	3e-101
Residual	−0.03	−0.0038	0.0012	−0.76	0.78

**Table 2:** Maximum likelihood parameter estimates, standard error, test statistics, and p-values from the spatial simultaneous autoregressive lag model (Eq. (1)).

Parameter	Estimate	Standard Error	T-statistic	P-value
rho	0.52	0.071	7.3	4.1e-13
(Intercept)	1.2e+04	1.2e+04	1	0.32
Mkts	8.5e+03	3e+03	2.8	0.0047
Cattle_Sales_Head	−0.008	0.026	−0.31	0.76
lag.Mkts	−6e+03	7e+03	−0.86	0.39
lag.Cattle_Sales_Head	0.16	0.059	2.8	0.0057

**Fig. 2:**
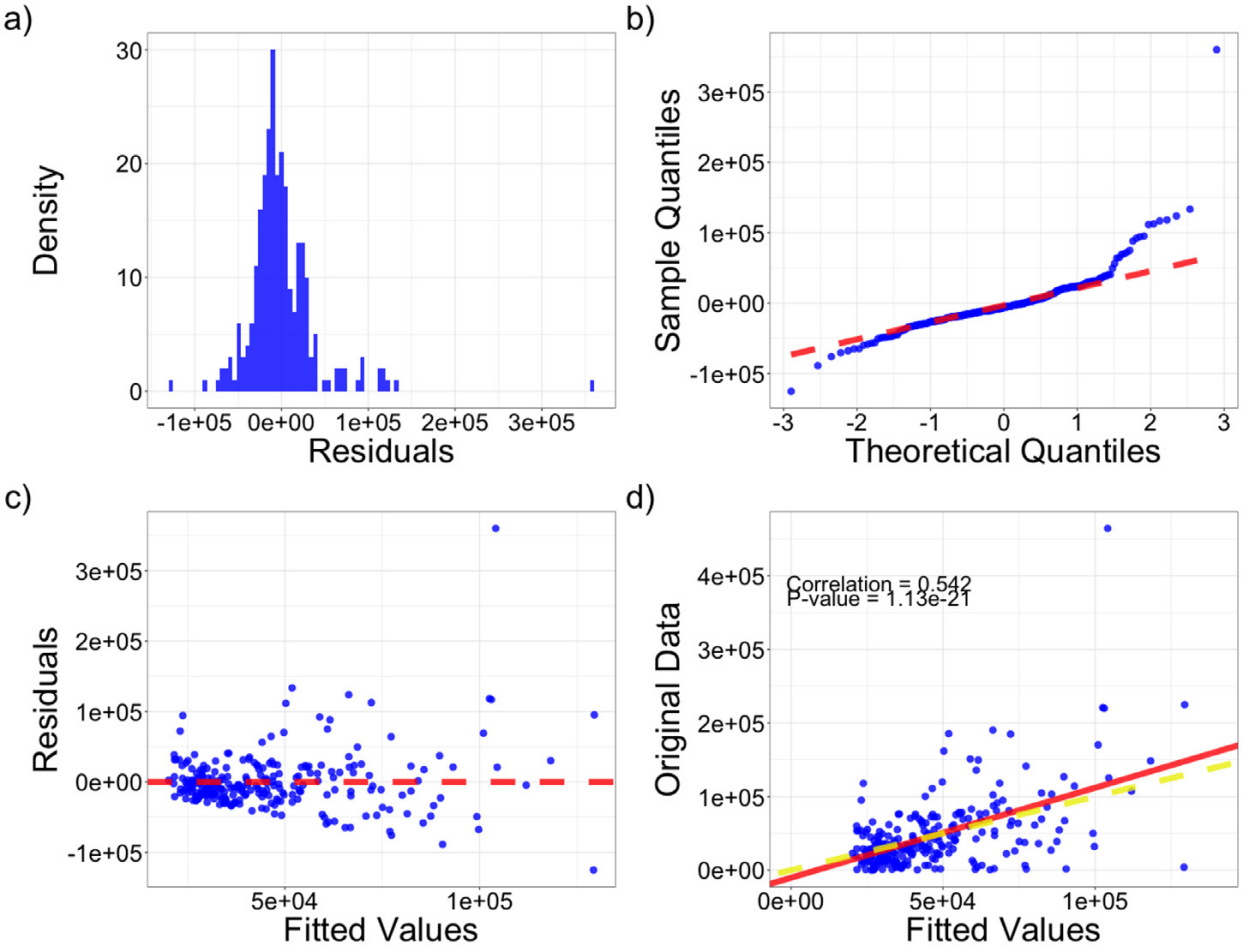
(a) Density plot of residuals from the simultaneous autoregressive lag model. (b) Quantile-quantile plot of residuals from the simultaneous autoregressive lag model. Red line represents the 1:1 line that indicates perfectly correlated theoretical and sample quantiles. (c) Residuals plot showing the relationship between predicted values from the model (fitted) and the residuals. The horizontal dashed line represents zero difference between fitted and observed values or perfect prediction. (d) Plot of model original data against model predictions. The yellow dashed line is a 1:1 line that represents perfect prediction and the solid red line is the line of best fit.

**Fig. 3:**
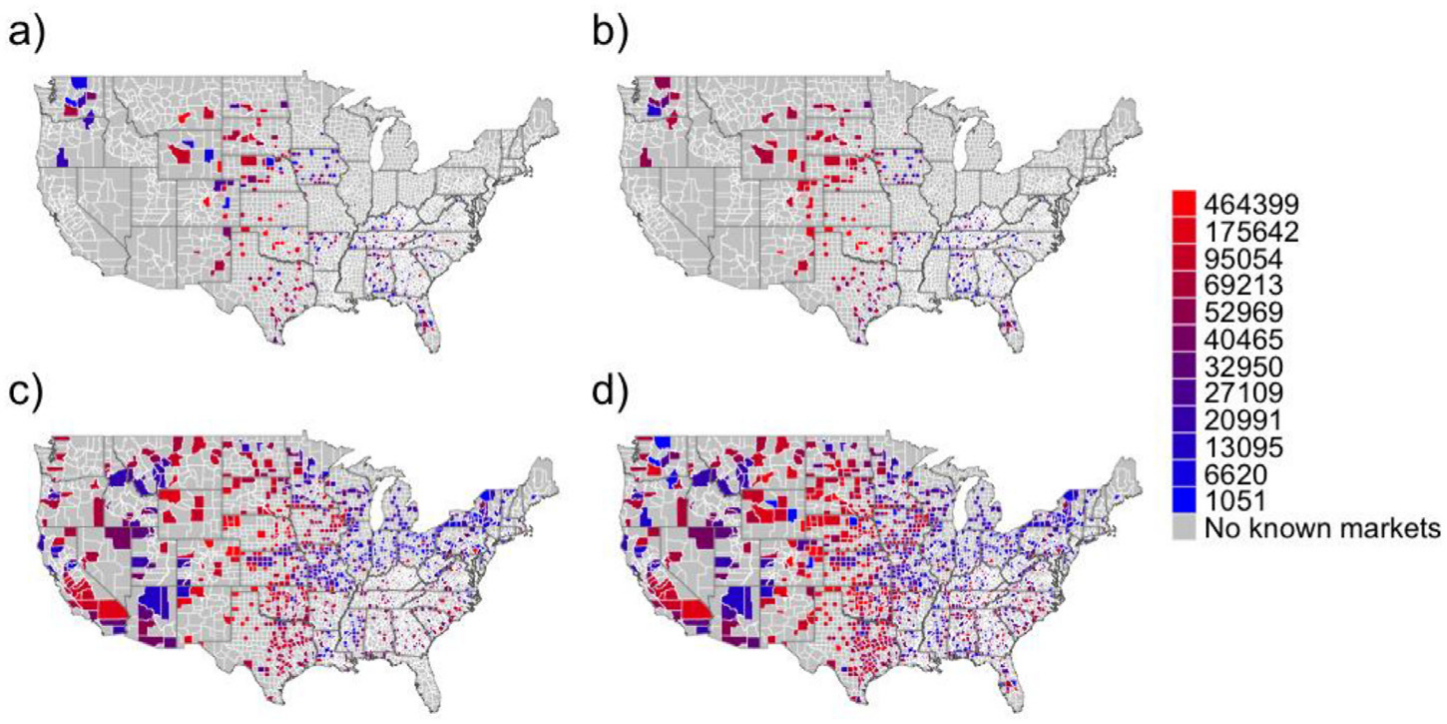
Distribution of mean annual market volumes at the county scale. Warm colors indicate larger volumes and cool colors smaller volumes. (a) Observed county-level market volumes only where data were available. (b) Predicted market volumes for counties where data were available. (c) Predicted market volumes in counties where data were unavailable. (d) Market volumes for all counties with markets. Data are used where market volumes are known and predictions when unknown.

We evaluated the model’s fit to the county-level market data by examining residuals as well as the correlation and similarity of spatial patterns between observed and fitted values. Residuals from the model fit were normally distributed, but the model failed to capture extreme values (Fig. 2a, b). Residual variance was also not constant (Fig. 2c). However, fitted values from the model were strongly correlated with the original dataset (R^2^ = 0.54, *p* < 0.001, Fig. 2d), and residuals lacked evidence of spatial structure (Moran’s *I* = −0.030, *p* < 0.0001, Table 1). Fitted values maintained spatial structure present in observed values (Moran’s *I* = 0.76, *p* < 0.0001, Table 1). Predicted county-level market sales (Fig. 3b) displayed very similar spatial patterns to observed county-level market sales used to fit the model (Fig. 3a), so we felt comfortable keeping number of markets and annual cattle sales as the only model covariates.

We used the model fit to predict market sales volumes in the 865 remaining counties that lacked volume data but contained markets (Fig. 3c). The final dataset includes original county-level volume data where preexisting data were available and modeled volume estimates where data were not available (Fig. 3d).

We used the *lagsarlm()* function from the R package *spdep* to fit Eq. (1) to average annual county sales, and the *predict.sarlm()* function to estimate unknown county sales [3,16].

## Ethics Statements

The authors declare that they have no known competing financial interests or personal relationships that could have appeared to influence the work reported in this paper.

## CRediT Author Statement

**Samuel M Smith:** Visualization, Writing – Original Draft, Writing – Reviewing and Editing**, Clayton Hallman:** Data curation, Investigation, Methodology, Validation, Formal analysis**,** Visualization, Writing – Reviewing and Editing**, Tom Lindström:** Supervision, Funding acquisition, Project administration**, Ryan S Miller:** Supervision, Funding acquisition Project administration**, Lindsay M Beck-Johnson:** Supervision, Funding acquisition, Project administration, Writing – Reviewing and Editing, **Stefan Sellman:** Supervision, Funding acquisition, Project administration, Writing – Reviewing and Editing**, Colleen T Webb:** Investigation, Methodology, Supervision, Funding acquisition, Project administration, Writing – Reviewing and Editing

## Data Availability

DryadData from: United States cattle market location and annual market sales estimate data (Original data)
